# Probing three-dimensional surface force fields with atomic resolution: Measurement strategies, limitations, and artifact reduction

**DOI:** 10.3762/bjnano.3.73

**Published:** 2012-09-11

**Authors:** Mehmet Z Baykara, Omur E Dagdeviren, Todd C Schwendemann, Harry Mönig, Eric I Altman, Udo D Schwarz

**Affiliations:** 1 Department of Mechanical Engineering and Materials Science, Yale University, New Haven, CT 06520, USA; 2 Center for Research on Interface Structures and Phenomena (CRISP), Yale University, New Haven, CT 06520, USA; 3 Department of Mechanical Engineering, Bilkent University, Ankara 06800, Turkey; 4 Physics Department, Southern Connecticut State University, New Haven, CT 06515, USA; 5 Physikalisches Institut at the Center for Nanotechnology (CeNTech), Westfälische Wilhelms-Universität, 48149 Münster, Germany; 6 Department of Chemical and Environmental Engineering, Yale University, New Haven, CT 06520, USA

**Keywords:** atomic force microscopy, force spectroscopy, NC-AFM, three-dimensional atomic force microscopy, tip asymmetry, tip elasticity

## Abstract

Noncontact atomic force microscopy (NC-AFM) is being increasingly used to measure the interaction force between an atomically sharp probe tip and surfaces of interest, as a function of the three spatial dimensions, with picometer and piconewton accuracy. Since the results of such measurements may be affected by piezo nonlinearities, thermal and electronic drift, tip asymmetries, and elastic deformation of the tip apex, these effects need to be considered during image interpretation.

In this paper, we analyze their impact on the acquired data, compare different methods to record atomic-resolution surface force fields, and determine the approaches that suffer the least from the associated artifacts. The related discussion underscores the idea that since force fields recorded by using NC-AFM always reflect the properties of both the sample and the probe tip, efforts to reduce unwanted effects of the tip on recorded data are indispensable for the extraction of detailed information about the atomic-scale properties of the surface.

## Introduction

Experimentally obtained information about atomic-scale interactions of specific surfaces with atoms, molecules, and other surfaces in their vicinity is crucial for a number of important scientific fields, including catalysis, thin-film growth, nanoscale device fabrication, and tribology, among others [1]. Shortly after the first atomic-resolution images of surfaces were obtained by noncontact atomic force microscopy (NC-AFM) [2–3], the method of dynamic force spectroscopy (DFS) was introduced, empowering experimentalists to characterize the tip–sample interaction in terms of normal forces *F*_n_, potential energies *E*, and the distance *z* between the tip apex and the sample surface [4–7]. More recently, thanks to improvements in the design of atomic force microscopes [8–9] as well as the development of new data-acquisition strategies [10–11], DFS measurements have been extended to two and three spatial dimensions. As a result, tip–sample interaction forces and energies can be measured as a function of both the tip–sample distance *z* and the lateral position (*x*, *y*) of the tip apex above the sample surface. Force fields have now been recorded on NiO(001) [10,12–13], MgO/Ag(001) [14], NaCl(001) [15–16], Si(111)-(7×7) [17–19], HOPG [20–21], KBr(001) [9,22–23], Cu(111) [24], and CaCO_3_(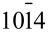) [25] surfaces, as well as single molecules of PTCDA [26–27], pentacene [28], CO [29], C_60_ [30], naphthalocyanine [31], and individual carbon nanotubes [32–33]. Moreover, differentiating the tip–sample interaction energy data in the lateral (*x*, *y*) directions has enabled the determination of atomic-scale lateral forces experienced by the probe tip [12]. From such data, the forces required to manipulate single atoms and molecules laterally on sample surfaces were quantified [34] and the lateral force field on graphite could be studied in detail [20]. Finally, three-dimensional force spectroscopy experiments performed in a liquid environment have revealed the spatial distribution of water molecules at a water–mica interface [35].

**Figure 1:**
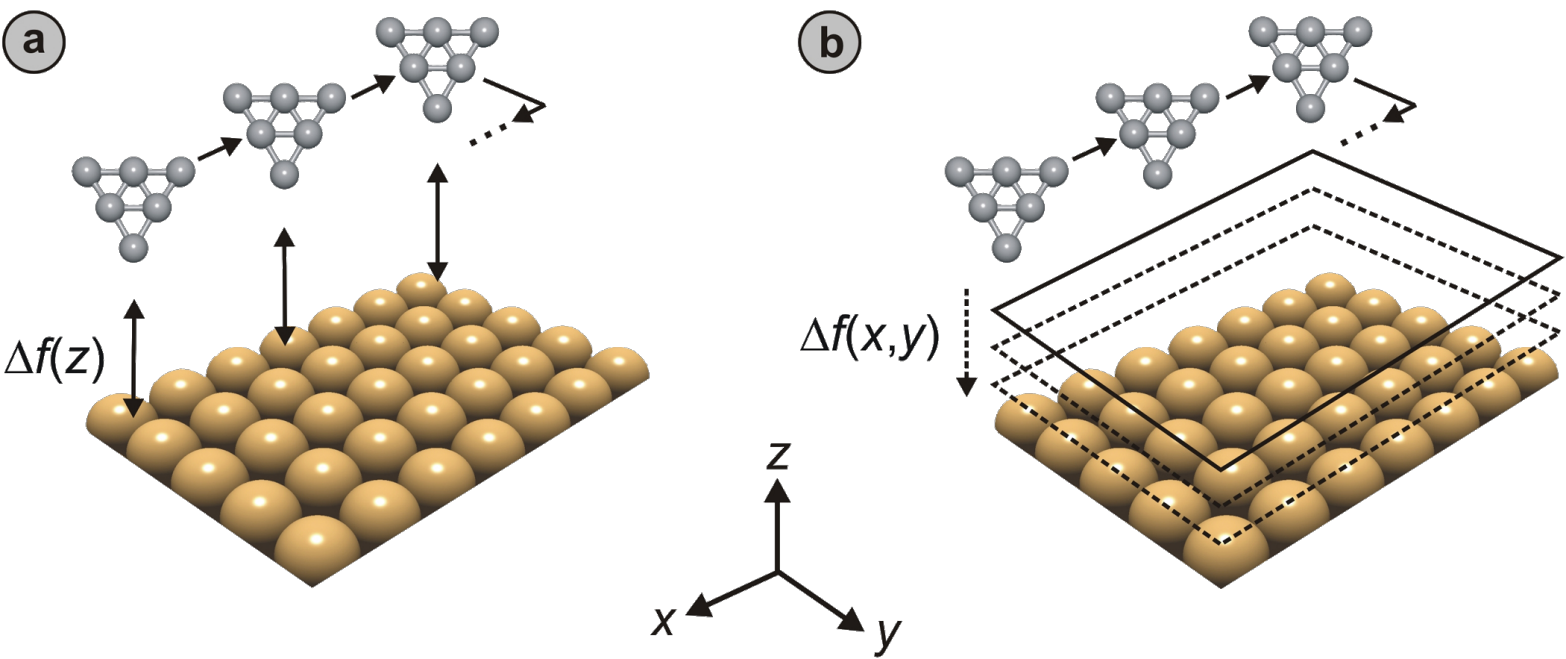
Schematic drawings illustrating data-acquisition procedures employed to record the atomic-scale surface force fields *F*_n_(*x*, *y*, *z*) experienced by a probe tip. While the *curve-by-curve* approach (a) relies on sequential recording of individual ∆*f*(*z*) curves at each (*x*, *y*) location on the surface that should be covered, the *layer-by-layer* approach (b) involves the consecutive recording of individual NC-AFM images at varying tip–sample distances *z*. In both cases, the resulting ∆*f*(*x*, *y*, *z*) array is converted to *F*_n_(*x*, *y*, *z*) data after data acquisition has been completed.

The methods most frequently reported in the literature to record two- and three-dimensional force fields above sample surfaces may be divided into two general categories (Figure 1):

1) The *curve-by-curve* method, in which individual curves of frequency shift versus tip–sample distance (∆*f* versus *z*) are recorded at a number of (*x*, *y*) locations on the sample surface and then combined to form full three-dimensional ∆*f*(*x*, *y*, *z*) arrays that are later converted to *F*_n_(*x*, *y*, *z*) and *E*(*x*, *y*, *z*) data [12,15–16,19,22–23,25–28,30–32,36].

2) Alternatively, the three-dimensional ∆*f*(*x*, *y*, *z*) array may be recorded *layer-by-layer*, by combining a series of *topographical* or *constant-height* NC-AFM images that contain ∆*f*(*x*, *y*) information for certain tip–sample distances *z* [9,11,20,23–24]. A subset of this method involves recording the frequency shift along a single line as *z* is varied (*line-by-line* recording). This yields two-dimensional cuts of ∆*f*(*x, z*), which may be later converted to *F*_n_(*x*, *z*) and *E*(*x*, *z*) maps [18,29,34].

Regardless of the data-acquisition method, there are many reasons why data sets acquired on identical surfaces may vary significantly, both quantitatively and qualitatively, including:

- thermal and electronic drift during the measurement,
- nonlinearities and creep associated with piezoelectric scan elements used in the microscope,
- variability of tip-apex structure and chemistry between different experiments, and
- elastic deformations of the tip under the influence of the surface force field.

The intent of this paper is to provide a comprehensive discussion of all the major limitations intrinsic to three-dimensional force spectroscopy by scanned probes that have to be considered during data interpretation. To that end, the effect that each of the four items has on the recording of atomic-scale surface force fields is analyzed, and it will be shown that the four factors may be best alleviated by combining specialized data-recording schemes with post-acquisition correction procedures.

## Results and Discussion

The goal of any microscopy technique is to obtain information on the investigated sample with as little ambiguity, uncertainty, and irreproducibility due to technique-inherent practical limitations as possible. In the case of NC-AFM, drift, piezo nonlinearities, and piezo creep result in an apparent spatial misalignment and distortion of characteristic image features compared to the true structure and location of the surface sites that induce them; elastic deformations of the probe tip can cause a lateral shift of features in data acquired at different heights; and tip asymmetry effects may further complicate the assignment of characteristic features observed in images, to actual sites on the sample surface. Finally, we need to consider that unavoidable variations in the tip-apex structure for independent measurements result in further irreproducibility. The first part of this section covers an in-depth analysis of the related issues, while the second part applies the findings to determine the optimum strategies for extracting reliable information on atomic-scale chemical and physical properties of sample surfaces.

### Part I: Artifacts in force-field spectroscopy measurements

#### Drift

Virtually all atomic-scale scanning probe microscopy (SPM) experiments suffer from unwanted relative movement of sample and probe tip with respect to each other during imaging and force spectroscopy, as a result of thermal fluctuations and the difference in thermal expansion coefficients of the building blocks of scanning probe microscopes. Considering that the recording of dense data arrays of frequency shifts above sample surfaces in vacuum usually takes several hours [20,28], the associated imaging/spectroscopy artifacts are especially problematic for force-field measurements performed at room temperature [25], which often feature lateral drift rates of angstroms per minute. In contrast, performing the experiments at low temperatures can *suppress* thermal drift to as little as a few angstroms per day [8].

An elegant approach to *correct* the effects of thermal drift in lateral directions during SPM imaging involves the use of atom-tracking and feed-forward positioning methods. Atom tracking [37] comprises the determination of the drift vector by measuring the shift in the position of an individual maximum in subsequent SPM images followed by an appropriate correction of the tip location that compensates for this drift. In contrast, the feed-forward procedure [17] is based on the real-time correction of drift during data acquisition by applying appropriate voltages to the scan piezo, which are calculated based on the assumption that the drift vector can be adequately predicted based on prior measurements. The two approaches have been successfully implemented in the past to measure both two- and three-dimensional surface force fields at room temperature and low temperatures on various sample surfaces [16,18–19,23,25,30]. One drawback is that, typically, frequent updates of the drift vector (as much as one atom-tracking measurement before the recording of each ∆*f* versus *z* curve in a *curve-by-curve* measurement [16,23]) are required due to the unpredictability of thermal drift and lack of control over temperature fluctuations. Thermal drifts leading to lateral displacements of the sample surface with respect to the probe tip, by more than one unit cell in the time required to collect an image, are also potentially problematic and in some cases limit *layer-by-layer* data acquisition to low temperatures [23].

An alternative approach to *x–y* drift correction involves manual post-data-acquisition shifting of images acquired by the *layer-by-layer* method [11]. In this approach, consecutive images that are part of the *layer-by-layer* dataset are laterally shifted against each other such that individual maxima in the images are aligned on top of one another. After all images in the dataset have been aligned accordingly, the (*x*, *y*) region common to all images is cut out and forms the basis for the ∆*f*(*x*, *y*, *z*) array that is later converted to interaction-force and energy data (*F*_n_(*x*, *y*, *z*) and *E*(*x*, *y*, *z*) arrays, respectively). With a sufficiently dense dataset consisting of images separated by only a few picometers in the *z* direction, gradual lateral shifts between subsequent images due to thermal drift may be precisely monitored and corrected for, provided that lateral drifts between images are significantly lower than one unit cell.

Thermal drift is, however, not limited to lateral displacements, but may also affect the accuracy of *z* values. Fortunately, several easy-to-apply procedures can eliminate the consequences of *z* drift on the measurements. For arrays compiled using the *curve-by-curve* method, such drift-induced distortions can be corrected by using standard line- or plane-fit algorithms. When acquiring data *layer-by-layer*, the necessary adjustments can be carried out by comparison with site-specific high-density ∆*f*(*z*) calibration curves recorded directly before and/or after the individual layers needed to assemble the actual data array. Alternatively, curves of tunneling current versus distance can serve the same purpose if the tunneling current, which is recorded together with the frequency-shift data, does not decay too fast to provide accurate calibration at all distances covered by the 3-D set.

A completely different source of drift may originate from the use of analog electronics for oscillation detection and amplitude/phase-feedback control during NC-AFM operation. With such setups, the output voltage that is supposed to faithfully reflect the cantilever resonance frequency may shift over time even if the resonance frequency stays constant. If this happens, the data acquisition software interprets the shift as an apparent change in the tip–sample distance, which it counteracts by adjusting the *z* deflection of the scan piezo. As a consequence, unwanted variations in the tip–sample distance are induced [12]. While such electronic drifts may be manually corrected for by checking the atomic corrugation values at various points during force-spectroscopy experiments, the use of digital electronics for NC-AFM detection and control generally eliminates the effects of electronic drifts on measured data.

#### Piezo nonlinearities and piezo creep

**Figure 2:**
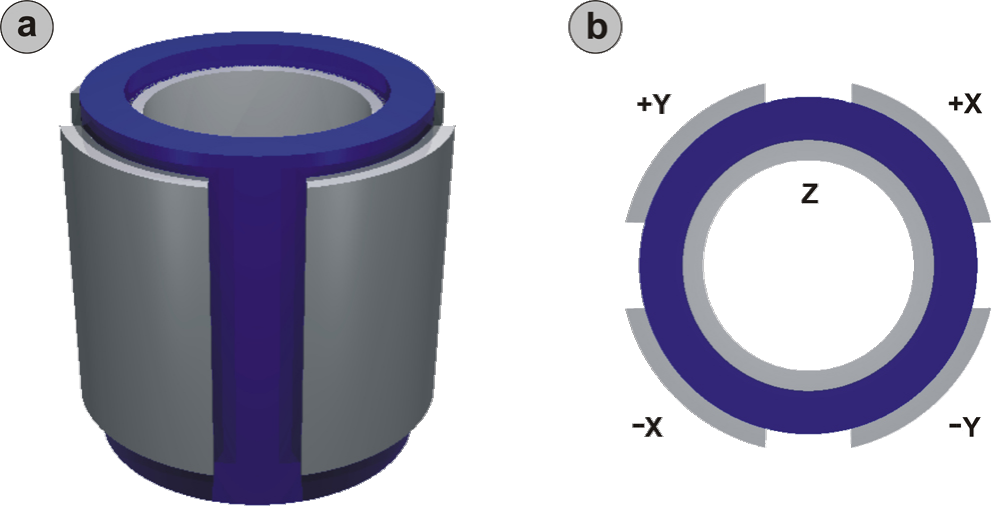
Isometric (a) and top (b) views of a piezoelectric scan tube employed as a precise positioning tool in scanning probe microscopes [38–39]. The tube itself (blue) is manufactured out of a piezoelectric material (usually lead zirconate titanate, PZT). Voltages applied to one of the four external metallic electrodes (grey; denoted with +X, −X, +Y, and −Y) are used to control the tube’s deflection in lateral directions, whereas the potential experienced by the inner electrode (denoted with *Z*) governs the tube’s vertical extension/contraction.

Positioning devices that employ piezoelectric materials to realize voltage-controlled relative positioning of the tip and sample are widely used in SPM experiments (see, e.g., Figure 2) [38–40]. Despite subpicometer positioning accuracy, piezoelectric scanners display fundamental shortcomings. The most important limitation originates from the fact that the relationship between applied voltage and the amount of extension/contraction undergone by the piezoelectric material is *nonlinear* as well as *time-* and *history-dependent* [40–41]. As a result, piezo scanners extend or contract less at the beginning of a scan line than at the end, which leads to hysteresis loops [40,42–43]. Similarly, piezo *creep* manifests itself as an additional, logarithmically decaying deformation of piezo elements after the application of a change in voltage [44]. Both phenomena implicate an uncertainty regarding the assignment of the specific voltages applied to the electrodes to an actual location (*x*, *y*, *z*) over the sample surface, which ultimately manifests as distortion in the recorded image, as well as in a finite difference between the actual physical positions of the scanner at the same voltage, between forward and backward scan lines.

To correct for these effects, commercial SPM equipment often employs strategies such as closed-loop scan elements that track the actual (*x*, *y*) position with deflection sensors in real time or the application of voltages in the form of distorted waveforms so that the resulting motion is linear with respect to voltage [38,45]. Allowing the piezotube to settle down for a certain amount of time after the recording of each curve/image during data acquisition, helps to reduce the influence of creep on the measured data further. If atomic resolution is achieved, apparent lattice distortions may be corrected after acquisition has been completed by using the known size, symmetry, and orientation of surface unit cells as input [46]. Another strategy is to experiment at low temperatures, where the effects of piezo nonlinearities, creep, and hysteresis are suppressed. Combined with the benefits regarding thermal stability discussed in the previous section, low-temperature data recording is found to be ideally suited for the reliable long-term recording of atomic-scale surface force fields [8].

For completeness, it should be noted that the limitations addressed above do not represent a complete list. Additional distortions of atomic-scale force fields may be caused, e.g., by a cross-coupling between the *x*, *y*, and *z* channels of the scanner due to either structural imperfections of the piezo elements or design-inherent coupling issues, or both. For the tube scanner in Figure 2, such structural imperfections may be due to small variations in the thickness of the tube walls, or due to restrictions in the ability of the tube to flex, which are imposed by the soldering or glue spots that are used to contact the electrodes; an example of a design-inherent issue is the tube sweeping out an arc when moved in lateral directions, which makes some distortion in *z* unavoidable. Another source of distortions may be due to small deviations of the scanner axes from the actual *x*, *y*, and *z* directions caused by incorrect alignment during microscope construction, where even differences of a few degrees may result in appreciable lateral shifts between tip and sample as the piezo material is deformed.

#### Tip-apex structure and chemical identity

Numerous theoretical and experimental studies have shown that the atomic-scale contrast in NC-AFM measurements is heavily dependent on the local structure of tip apices employed in the experiments, as well as on the chemical identity of the apex atoms (see, e.g., [28,47–55]). While, for specific cases, the contrast-formation mechanism may be explained by using a relatively simple picture of tip-apex polarity [47,54–55], generally more complicated tip-apex models and theoretical considerations need to be taken into account to understand the full effect of tip structure and chemistry on NC-AFM measurements [49–51]. Controlling the chemical identity of the probe tip employed in NC-AFM experiments down to the last few atoms of the tip apex has proven to be extremely difficult in the past due to oxidation issues associated with traditional Si cantilevers, as well as with metallic tips prepared by electrochemical etching, in addition to frequently observed tip changes that may lead to a modification of the tip apex on the atomic scale [56]. A notable exception presents itself in the form of metallic tip apices terminated by single molecules that have been deliberately picked up during SPM experimentation at low temperatures [28]. This approach, which has been previously employed in scanning tunneling microscopy imaging [57–58], has recently been applied to NC-AFM imaging and force spectroscopy experiments with great success [28,31]. As surface force fields recorded with such well-defined tips provide useful information about the interaction of the attached molecule with the probed surfaces, the application of molecule-terminated tips is expected to become more and more popular in force-spectroscopy experiments [1]. Lastly, let us note that a recently reported alternative method to control tip-apex chemistry involves the in situ deposition of metallic thin layers on commercial Si cantilever apices [56]. In combination with theoretical calculations, maxima in NC-AFM images provided by such well-characterized tips on the ionic surface of NaCl(001) have been shown to unambiguously coincide with surface anions, facilitating atomic-scale chemical identification.

#### Tip elasticity

In addition to drift and piezo effects, the accuracy with which a numerical value, obtained through two- or three-dimensional force field spectroscopy, can be straightforwardly assigned from its apparent position (*x*, *y*, *z*) in the data array to an actual location relative to the sample lattice, is further limited by elastic deformations of the tip apex under the influence of external forces as it is scanned over the sample surface. This is because these deformations cause the tip apex to be at a different location than we assume it to be, which results in a distortion of the recorded force field. The extent of this distortion depends on the local strength of the tip–sample interaction force as well as on the lateral and vertical stiffness of the specific tip.

The effect of tip-apex deformations on dense force-field spectroscopy experiments has been previously analyzed in the literature [9,16,23]. In particular, Such et al. listed several criteria that allow the identification of tip-apex relaxation effects in NC-AFM-based force spectroscopy experiments [9]: (a) Lines showing rapid changes in two-dimensional, horizontal topography/frequency shift maps of the surface; (b) significant shifts in contrast patterns observed in such maps as the tip moves closer to the surface; (c) force-versus-distance curves exhibiting extended plateaus, over several hundreds of picometers, of more or less constant force close to the surface, as opposed to the expected onset of repulsive force; and (d) two-dimensional vertical force cuts where the force on several lattice sites becomes maximum at a certain height above the surface and stays constant until the plane of closest approach is reached, which is a direct consequence of (c). Additionally, Kawai et al. [16] and Fremy et al. [23] performed drift/creep-corrected three-dimensional force-field-spectroscopy experiments using atom-tracking, on NaCl(001) and KBr(001), respectively, in which shifts of characteristic maxima in atomic-scale images and significant distortions of the observed contrast patterns attributed to tip apex elasticity were observed as a function of tip–sample distance.

**Figure 3:**
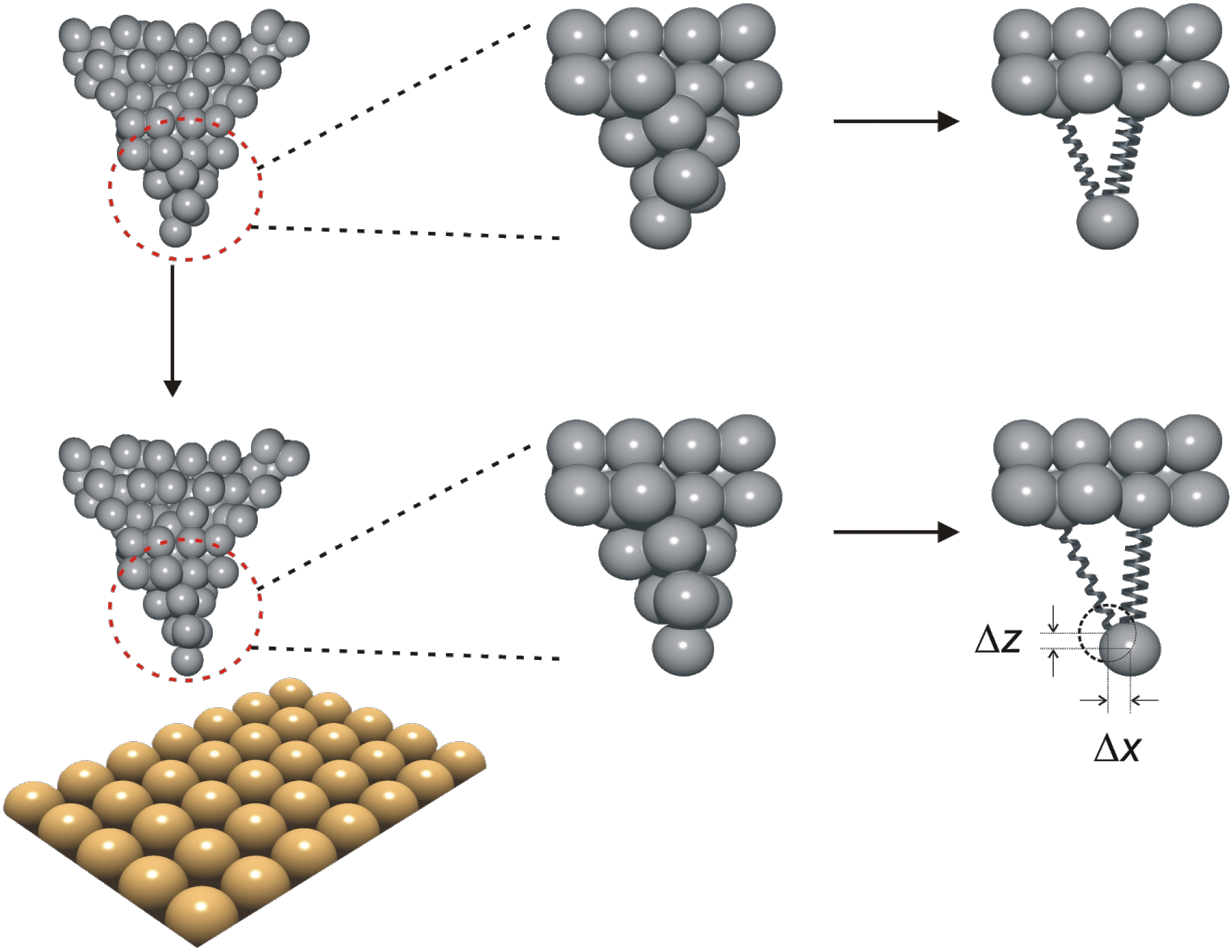
Schematic drawing describing the elastic deformation/bending of an asymmetric tip apex as the tip–sample distance is reduced. Top row, left: an asymmetric tip located away from any surface; middle: magnification of the tip apex; and right: a mechanical spring model that mirrors the elastic properties of the last tip atom. Note that due to the asymmetric character of the tip apex, the spring on the right is stiffer than the spring on the left. Bottom row, left: The tip is now located close to a surface, where it feels an attractive interaction in the *z* direction; middle: zoom onto the tip end; and right: equivalent mechanical model. The surface forces pull the last tip atom a distance Δ*z* towards the surface when compared to the location of the atom relative to the tip base in the top row (dashed circle). Since the right spring in the equivalent mechanical model is stiffer than the spring on the left, this motion also results in a net lateral displacement Δ*x*.

On our path towards finding strategies that reduce the impact of tip deformation on the recorded data, we start by noting that atom-tracking and feed-forward techniques, which have been successful in correcting for the effects of thermal drift and, at least partially, piezo effects, do not offer viable solutions. Next, we recognize that caution has to be exercised when analyzing data sets where relaxations lead to significant distortions in contrast patterns, because the associated data may be so heavily influenced by the properties of the probing tip that little useful information can be gained about the sample surface. To avoid misinterpretations, such measurements should be discarded. For the analysis of data acquired with tips that display only minor gradual contrast changes with distance, lateral tip apex bending may arise from two sources: (i) lateral forces inducing atomic-scale relaxations in the last few atomic layers of the tip apex, based on the local position of the tip above the surface, and (ii) normal (i.e., vertical) forces that will cause a bending and an effective overall lateral movement of the tip apex in a particular direction, which occurs if the tip used to probe the sample surface is asymmetric (Figure 3). Deformations due to (i) could be accounted for if the lateral stiffness of the apex were known, which it is usually not. However, if measurements are restricted to distances at which low site-specific lateral forces manifest, rough estimations of the expected deformations using typical values [59–60] suggest that they may be small enough to be ignored for all practical purposes. Deformations due to (ii), on the other hand, may be largely compensated by employing the post-data-acquisition correction procedures described earlier for the layer-by-layer approach [11].

#### Tip asymmetry

Even though imaging artifacts observed in atomic-scale scanning probe experiments are often associated with the use of asymmetric tips [16,61], a comprehensive understanding of the link between asymmetric tip geometries and the imaging artifacts they cause is still not complete. In this part of the paper, we present the highlights from a systematic study of the fundamental effects that asymmetric tips have on the measurement of atomic-scale surface force fields. The corresponding simulations, which use Matlab-based code [62], feature basic model geometries for tip and sample consisting of rigid atoms that interact through analytical potentials (both Lennard-Jones (L-J) and ionic). Even though these assumptions represent an oversimplification, as tip–sample contacts will relax upon tip approach and short-range interactions may differ substantially from those predicted by the potentials employed here, we still expect such simulations to provide valuable insights into the general trends that describe how tip asymmetry manifests in 3-D data sets.

For the computations, the Lennard-Jones potential between two atoms *V*_L-J_ was calculated by using

[1]
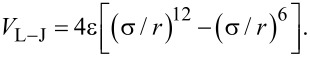


Here *r* denotes the distance between the centers of the two atoms, ε the depth of the potential well, and σ the finite distance at which the potential vanishes (note that σ = *d*/1.12, where *d* is the hard-sphere diameter of the atom). To obtain appropriate σ and ε values, the Lorentz–Berthelot mixing rules (σ_12_ = (σ_1_ + σ_2_)/2 and ε_12_ = (ε_1_ × ε_2_)^0.5^ [63]) were employed. In cases where the interacting atoms were ionized, a Coulomb potential *V*_C_

[2]
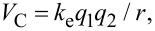


was added to the Lennard-Jones interaction with *k*_e_ reflecting the Coulomb constant, *q*_1_ and *q*_2_ the ionic charges, and *r* the distance between the ions. Total interaction potentials are obtained by summing up the individual potentials between each tip and substrate atom. The normal force *F*_n_ is then calculated by taking the derivative of the total interaction potential in the vertical direction.

**Figure 4:**
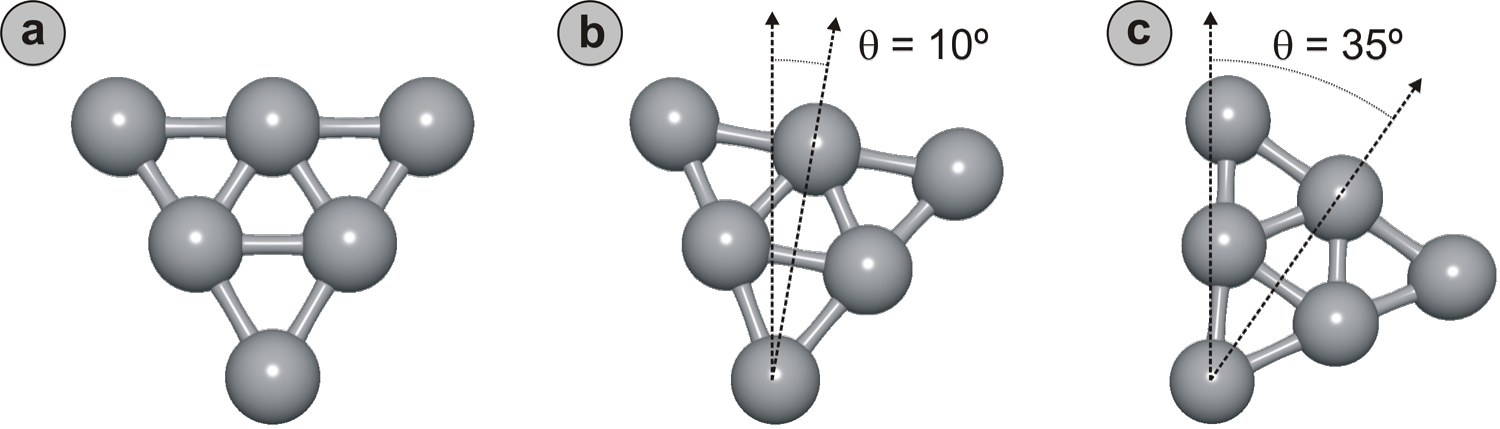
Planar three-layer tip apex models used in the analytical simulations, featuring close-packed atoms arranged with 2.70 Å nearest-neighbor distance (2.20 Å for ions with +1*e* charge). ε is 694 meV (680 meV if ionized) [64]. (a) Symmetric tip apex. (b) and (c) successively more asymmetric tip apices are obtained by rotating the tip model of (a) around the front-most atom with increasing angles θ.

The model tip apices used in the study are constructed from six close-packed atoms arranged in a three-layer planar configuration with the structural characteristics of a single Pt(111) plane (Figure 4a). The desired asymmetry in the tip-apex structure is obtained by rotating the model structures by an angle θ around the front-most atom (Figure 4b and Figure 4c). Even though we focus solely on this particular type of tip for the present discussion, we have calculated all cases for closed-packed planar tips featuring anything between a single atom and up to 15 atoms (five-layer 2-D tips), as well as for a full set of 3-D closed-packed tips with up to 25 atoms (five-layer 3-D tips). As a general rule, all trends displayed in the results below are more emphasized the more atoms are included in the tip, in particular for ionic interactions; but comparison also confirmed that the observed effects are representative for the overall behavior of asymmetric tips within the limited range of validity of this simple conceptual approach.

**Figure 5:**
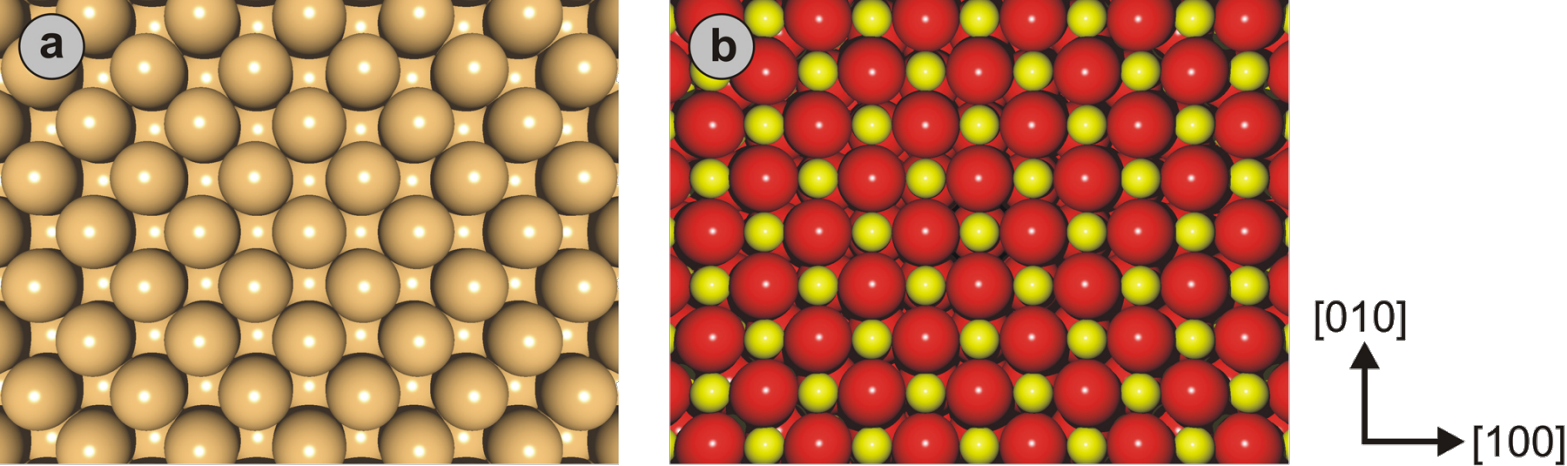
Illustrations of the model surfaces investigated in this section. (a) The Cu(001) surface in FCC configuration. Beige spheres represent individual copper atoms with a diameter of *d*_Cu_ = 2.60 Å and ε_Cu_ = 415 meV [64]. (b) The ionic NaCl(001) surface in which the large, red spheres are Cl^−^ ions with *d*_Cl−_ = 3.34 Å and ε_Cl−_ = 30.0 meV, and the small, yellow spheres are Na^+^ ions with *d*_Na+_ = 2.32 Å and ε_Na+_ = 15.4 meV. Crystallographic directions are indicated for both surfaces on the right.

Two different surfaces were investigated as part of the simulations described here (Figure 5): a surface that features all the structural characteristics of Cu(001), but interacts purely through L-J forces with the tip (i.e., we do not reproduce “true” metallic interactions, to keep calculations simple); and a surface with the structure of NaCl(001), where ionized atoms feel a Coulomb potential in addition to the ubiquitous L-J contribution. As is the case for the tips, substrate atoms were assumed to be immobile hard spheres, i.e., material relaxation effects are excluded. Surface cells were chosen to comprise 33 × 33 atoms with a thickness of five layers, as this cell size could be calculated quickly but was found to be large enough to avoid boundary effects.

**Figure 6:**
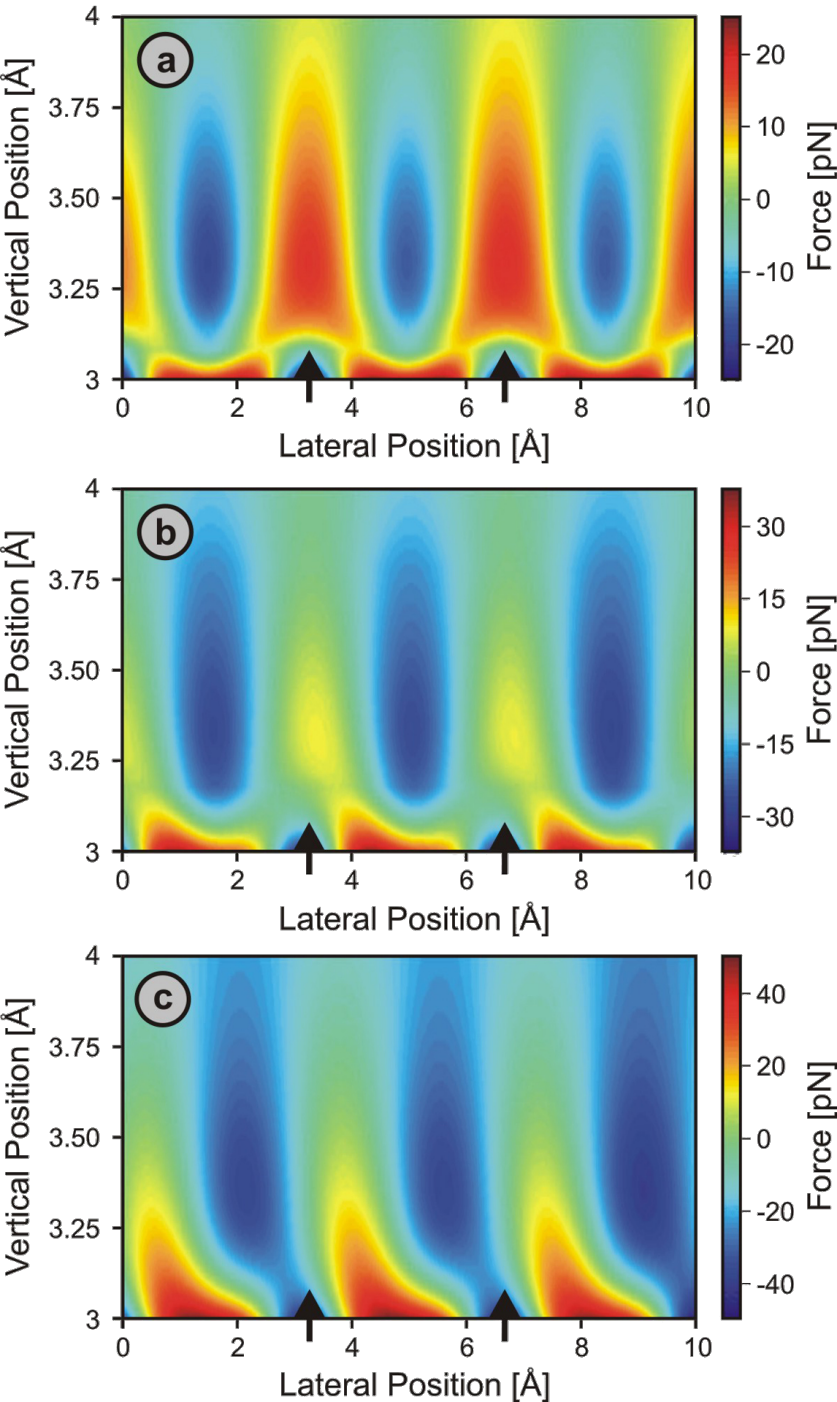
Simulated 2-D force fields over a Cu(001) surface along the [100] direction probed by symmetric (a: θ = 0°) as well as asymmetric (b: θ = 45°; c: θ = 58°) model tip apices that feature geometries as illustrated in Figure 4 while being assumed to interact solely through Lennard-Jones forces with the sample. The difference between the interaction force at each point and the mean interaction force at that height is displayed for better contrast visibility. The vertical position axis indicates the distance in the *z* direction between the center of the front-most tip atom and the *x*–*y* plane defined by the centers of the surface copper atoms. Black arrows mark the lateral positions of the surface Cu atoms.

To start, we focused on the Cu(001) surface using two-dimensional vertical cuts representing normal L-J forces along the [100] direction. When a symmetric tip is used (i.e., θ = 0°), it is observed that the individual force fields associated with surface atoms are *symmetric*, i.e., evolve in a *straight* configuration while moving away from the sample surface. Thereby, the force maxima are situated directly above the surface copper atoms (referred to as *atomic sites* in the following) for most tip–sample distances (Figure 6a). At very close separations, however, the atomically sharp tip apex employed in the simulations experiences a larger attractive force on the site of the minima of the surface potential (the *hollow sites*). This force contrast flip causes a crossing of the ∆*f*(*z*) curves recorded above the atomic and hollow sites similar to the one previously observed in simulations carried out for a Xe(111) surface [65], which in turn limits the closest distance at which constant-frequency-shift images can be recorded. Therefore, high-resolution 3-D force field measurements using the *layer-by-layer* approach with active feedback are likely restricted to tip–sample distances that are unaffected by contrast flips.

This situation changes if *asymmetric* tips are employed. For θ values of 45° and 58° (Figures 6b and Figure 6c, respectively), the simulated atomic-scale force fields become lopsided, especially in close proximity to the sample surface, where an increasing lateral offset between the locations of perceived force maxima and the (*x*, *y*) positions of the surface copper atoms develops with decreasing tip–sample distance. Similarly shaped force spectra have previously been reported for two-dimensional force-section measurements on the surface of graphite [21]. While an elastic deformation of the tip apex in the lateral directions during the measurements could be responsible for such effects, our simulations suggest that strongly asymmetric tips provide an alternate explanation for the observed patterns. We note that for θ = 58°, one side of the tip is almost parallel to the surface, which thus may represent an illustrative but extreme case for tip asymmetry under practical conditions.

The fact that the location of the maximum attractive force smoothly moves from the atomic to the hollow site upon decreasing the tip–sample distance is of particular concern when applying the *layer-by-layer* approach for data acquisition in combination with post-data-acquisition correction procedures. As the symmetry of the surface unit cells of atomic and hollow sites of the Cu(001) sample is identical, the gradual shifting of the force maxima from one to the other becomes indistinguishable from the effects of thermal drift or overall elastic bending of the probe tip. Considering that most tips employed in NC-AFM measurements are asymmetric to a certain extent, this experimental approach should thus be avoided for surfaces with such characteristics, or, at the very least, limited to regions sufficiently away from the sample surface. However, even though such complications can in principle be avoided by applying atom-tracking or feed-forward techniques, we note that their inherent inability to correct for elastic deformations of the tip apex jeopardizes the reliability of such real-time approaches to drift correction to the extent that it is unclear a priori which approach to drift correction, i.e., real-time or post-acquisition, is superior.

**Figure 7:**
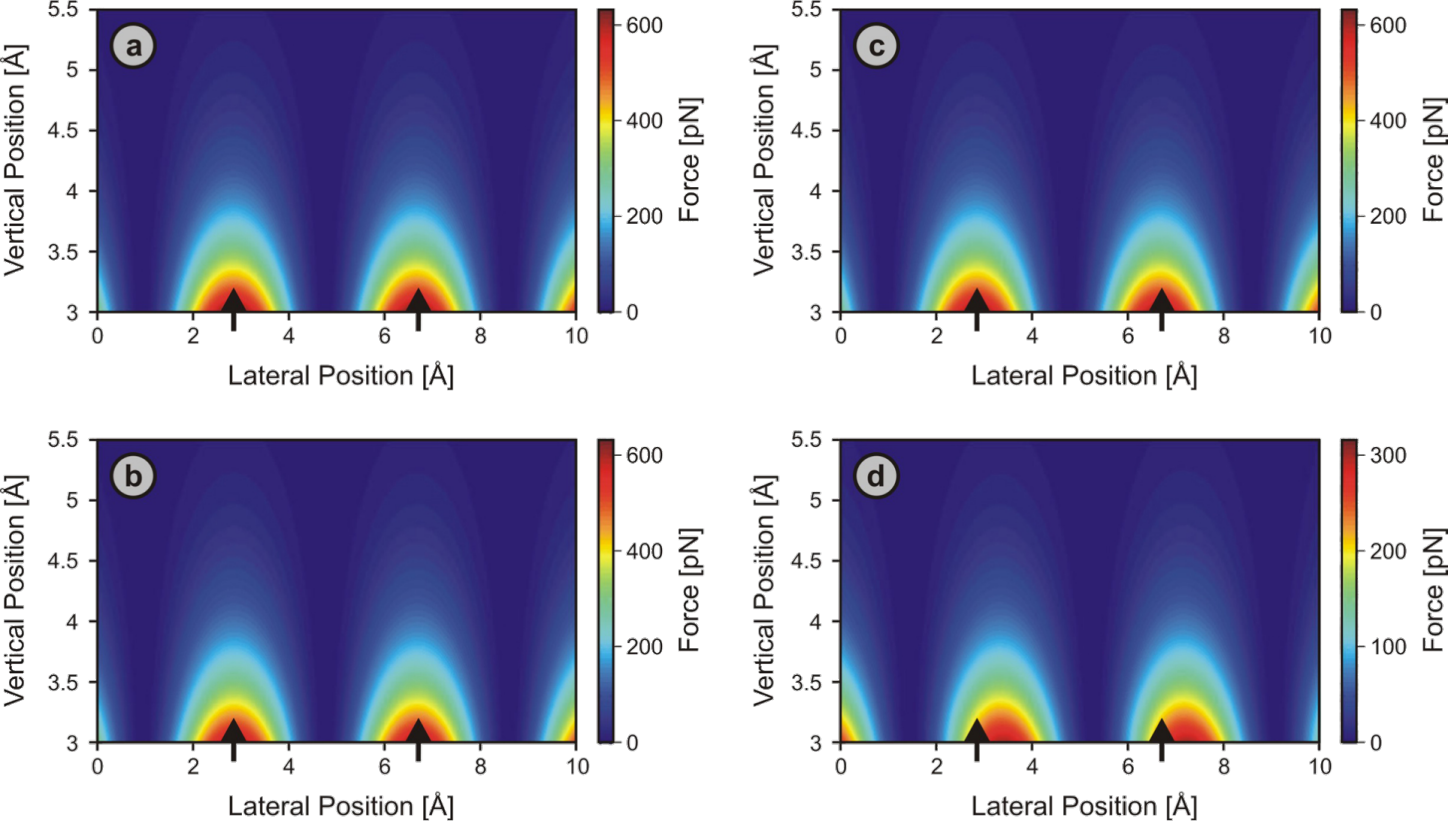
Simulated 2-D force fields over the NaCl(001) surface along the [110] direction probed by a symmetric (θ = 0°; a, c) and an asymmetric (θ = 56°; b, d) tip apex. In (a) and (b), only the front-most tip atom carried a trapped positive charge, while in (c) and (d) all atoms had been artificially ionized with +1 to explore the effect of charge distributions in asymmetric tips. In all panels, the difference between the absolute interaction force at each point and the minimum calculated force is displayed for better contrast. Black arrows mark the positions of the chlorine surface ions, which coincide with the force maxima in all cases but (d), in which the force maxima and the atomic positions are shifted by about 0.7 Å.

**Figure 8:**
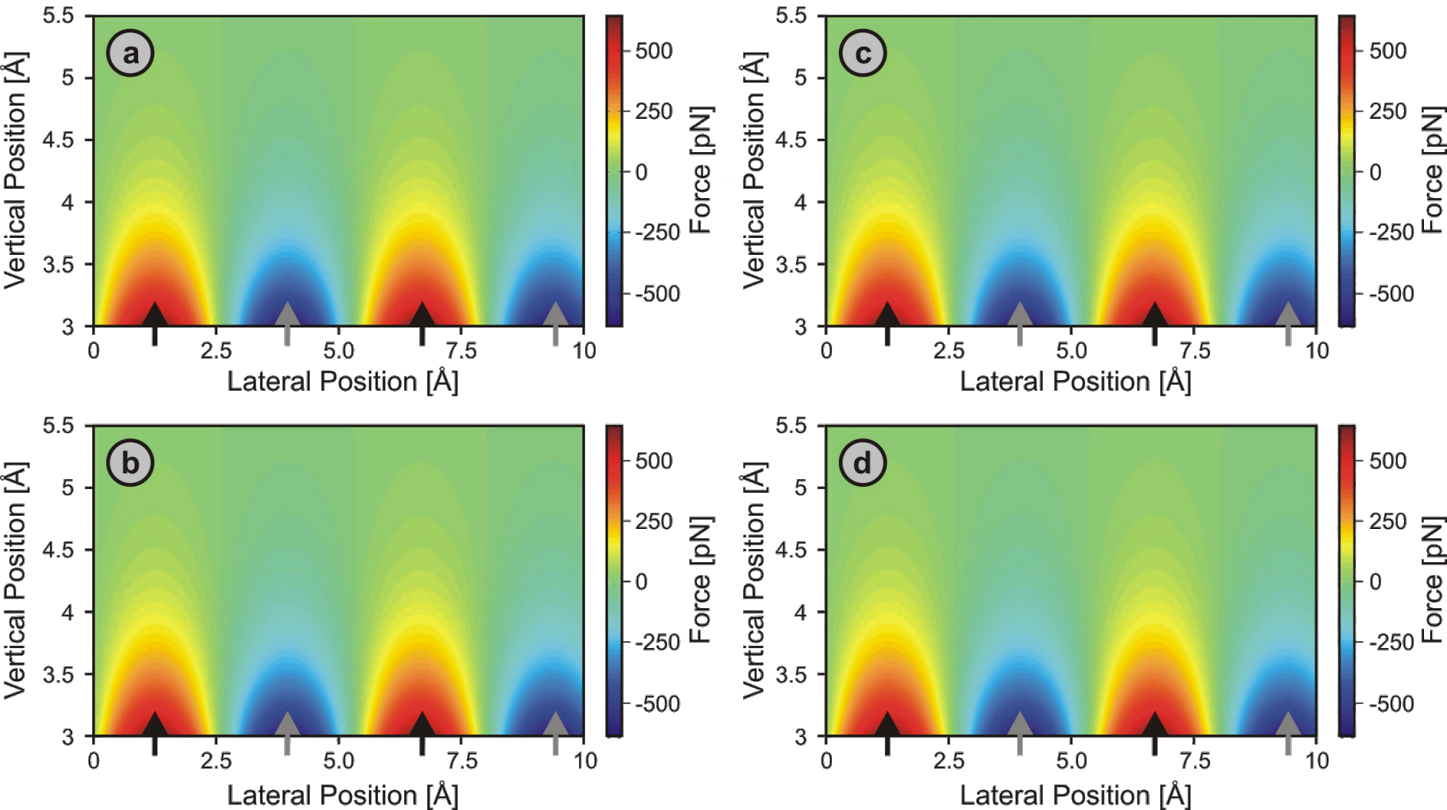
2-D force fields over the NaCl(001) surface simulated using parameters identical to the corresponding panels in Figure 7, but this time taken along the [100] direction. The difference between the absolute interaction force at each point and the mean calculated force is displayed for better contrast. In all cases, force maxima (red) coincide with the positions of the chlorine ions of the surface (indicated by the black arrows), while force minima (blue) concur with the sodium-ion lattice sites.

The effects of tip asymmetry on simulated force spectra acquired on ionic surfaces are inherently different from the simulation results obtained for Cu(001), as we will see below with the case of the NaCl(001) surface. Towards this end, we first outfit our planar six-atom tip as displayed in Figure 4 with a trapped positive, unity charge localized at the center of the front-most atom, which reflects a reasonable way of representing a charged tip [48]. The findings are then compared to the case where each of the atoms features a single trapped unity charge. Though this situation would be unrealistic for a real platinum tip, it allows us to get a feel for the trends that charge distributions in tips may impose on image contrasts. Figure 7 shows the results obtained along the [110] direction, which includes Cl^−^ ions only, while results along the [100] direction, which features both Cl^−^ and Na^+^ ions, are displayed in Figure 8.

We find that the locations of the force maxima coincide with the actual positions of the chlorine surface atoms in all cases except for Figure 7d, in which we observe an absolute lateral shift. But even in this instance, the individual force-field spectra associated with surface atoms evolve in a straight configuration, as the type of lattice site responsible for maximum forces (the Cl^−^ ions) does not change throughout the simulated height regime, in contrast to the simulations with L-J force only, performed on Cu(001). The reason for this difference lies in the distance dependence of the underlying atomic potentials. Since the ionic potential dominant in simulations on NaCl(001) has a long-range character as opposed to the L-J potential, the force field spectra are not as sensitive to the local changes in tip-apex structure induced by tip asymmetry for the majority of simulated heights. Perhaps more interestingly, the effect of asymmetric tip apices on simulated force spectra is nearly absent in the [100] direction for all investigated tips (Figure 8). The underlying reason is that the [100] direction on the sample surface includes both Cl^−^ and Na^+^ ions, and the additional attractive forces experienced by a *right-leaning* tip due to Coulombic interactions with the Cl^−^ ions are cancelled out by an equal increase in the amount of repulsive interactions with the Na^+^ ions of the surface, indicating that asymmetry effects in force spectra over ionic surfaces are strongly direction-dependent.

To summarize this section, we have found that:

1. For long-range interactions, force fields evolve straight into the space above the surface, even with asymmetric tips, because there is not much change in the relative contribution of individual tip atoms to the total tip–sample interaction with distance. Therefore, force spectroscopy experiments may be reliably performed and interpreted at all distance regimes, and post-data-acquisition correction procedures in conjunction with *layer-by-layer* data acquisition may be employed. With the example of ionic surfaces and Coulomb-force-dominated tip–sample interactions, it was found that the actual lattice sites responsible for the force maxima in the attractive tip–sample interaction regime (i.e., Cl^−^ ions in the case of NaCl(001) and a positively charged tip) coincide with the perceived force-maxima locations, except for in the case of very asymmetric tips featuring charge distributions (multiple localized charges of the same sign) at the apex, in which a lateral shift between force maxima and the locations of the surface ions occurs.
2. In contrast, when interactions between individual tip and sample atoms are sufficiently short-ranged, the lattice site exhibiting the most attraction on the front-most tip atom may move from an atomic to a hollow site upon tip approach. In such a case, tip asymmetry may lead to distortions in force fields, with the force maximum experienced by the tip as a whole smoothly moving from being near the atomic site towards being near the hollow site with decreasing tip–sample distance, as illustrated in Figure 6. Considering that most tips employed in NC-AFM measurements are asymmetric, 3-D force-mapping experiments performed on such samples may be instructive only for large enough tip–sample distances. If smaller distances are included in the analysis, post-data-acquisition correction procedures should be generally avoided if the effects of tip asymmetry cannot readily be distinguished from thermal drift or lateral shifting due to overall elastic bending of the probe tip.
3. For both short-range and long-range interactions, force-field characteristics associated with individual surface atoms on defect-free surfaces exhibit a *straight* and *symmetric* nature when probed with *symmetric tips* consisting of *immobile, hard, sphere-like atoms*. Thus, any experimentally observed deviations from this straight, symmetric character are necessarily due to either tip asymmetry or the elastic properties of the tip, or a combination of both.

### Part II: Comparison of data-acquisition procedures for atomic-scale force field spectroscopy

To help facilitate a reliable interpretation of 3-D force field data, we will summarize in this section the key points that have to be considered for selected experimental approaches. Thereby, we will focus on the following three methods, as they have been the main methods reported in the literature so far:

- *Layer-by-layer* data acquisition at low temperatures with post-data-acquisition correction procedures, referred to in the following as “method A” [11,20];
- *Curve-by-curve* data acquisition, mostly at room temperature, with atom-tracking/feed-forward procedures (“method B”) [16,19,23,25,30];
- *Curve-by-curve* or *line-by-line* data acquisition at low temperatures, involving the use of a *reference image* for drift correction at set time intervals (“method C”) [28,31].

Table 1 summarizes to what extent the methods listed above address the specific experimental issues discussed earlier. Note, however, that other combinations of experimental and data analysis procedures are possible, and experimentalists can tailor the exact approach to represent the best combination of experimental capabilities, post-acquisition processing, and artifact avoidance.

**Table 1:** Overall comparison of methods for atomic-scale force field spectroscopy. Items marked with 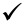 are satisfactorily resolved, while entries labeled with 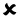 remain to be addressed.

	method A	method B	method C

**thermal drift**	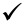 low thermal drift corrected post-acquisition	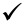 data acquisition has to be interrupted frequently to correct drift	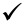 data acquisition has to be interrupted to correct drift
**piezo nonlinearities and creep (*x*–*y* plane)**	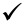 reduced piezo effects can be corrected post-acquisition	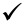 remaining piezo effects can be corrected post-acquisition	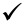 reduced piezo effects can be corrected post-acquisition
**variability of tip structure/chemistry**	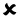 tip changes easily detectable during data acquisition	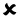 tip changes may not be visible during data acquisition	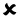 tip changes may not be visible during data acquisition
**tip elasticity**	~ 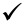 minor tip elasticity effects can be eliminated post-acquisition	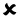	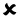
**tip asymmetry**	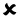 may cause problems for post-acquisition drift correction on certain surfaces	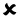	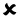
**additional notes**	contrast distortions readily detectable during data acquisition	irreversible tip changes much more likely at room temperature	–

From Table 1, we see that all three methods are able to satisfactorily account for the effects of thermal drift and piezo nonlinearities/creep. The variability of the tip-apex structure and chemistry between different experiments remains an inherent problem associated with NC-AFM, and efforts to obtain well-defined tips (such as the deliberate adsorption of a CO molecule on the tip apex; see [28]) are expected to be utilized in an increasing number of experiments, regardless of the specific methods used to perform the force spectroscopy. The effect of tip asymmetry on recorded force spectra is also an inherent tip-related problem that is especially critical for surfaces where the tip–sample interactions responsible for atomic resolution are predominantly short-range. Even though none of the methods above can eliminate the effect of tip asymmetry on the recorded force spectra, the application of post-data-acquisition shifting of subsequent images in method A could lead to misleading results on surfaces where the force maxima shift from one lattice site to another with indistinguishable symmetries. However, as long as tip–sample interactions are predominantly electrostatic in nature (e.g., on ionic crystals such as NaCl and KBr, as well as most metal oxides), method A holds a notable advantage over the other methods, as it allows the correction of lateral shifts in force maxima due to overall elastic deformations/bending of an asymmetric tip apex with increasing external normal forces at decreasing tip–sample distance.

While the post-data-acquisition correction procedures employed in method A do not allow researchers to distinguish between the exact source of such lateral shifts (which may be either caused by drift, creep, or elastic bending of asymmetric tips due to normal forces) [23], they nevertheless correct for such effects to a certain extent. Post-data-acquisition drift correction is best suited for low-temperature experiments, since (i) the drift rates are low enough that lateral shifts between consecutive images are small (significantly less than the lattice constant of the sample surface in question); (ii) thermal drift is often not random, but gradual, as the microscope temperature asymptotically approaches the equilibrium temperature of the cryogen [11]; and (iii) gradual changes in contrast (such as the appearance of stripe-like features between force maxima [20]) are clearly observable using sufficiently dense datasets. On the other hand, it is unsuitable for datasets in which contrast patterns alter so much with changing tip–sample distance that the alignment of characteristic maxima in subsequent images becomes problematic. However, no current method allows one to account for the effects of local, site-specific lateral forces causing such atomic-scale elastic relaxations in the tip apex. Lastly, it should be pointed out that one aspect where *layer-by-layer* acquisition of frequency-shift data proves especially advantageous, as opposed to *curve-by-curve* recording, is that distance-dependent distortions in the measured tip–sample interaction due to tip asymmetry and/or elastic relaxations manifest clearly during data acquisition as contrast changes, whereas in the *curve-by-curve* data-acquisition strategy such effects generally become only observable after data processing.

## Conclusion

Various methods and procedures employed to measure atomic-resolution surface force fields by NC-AFM have been reported and compared with respect to the extent to which they address issues such as drift and piezo nonlinearities, as well as tip-related problems of asymmetry and elasticity. While drift and piezo creep may be addressed in a number of ways, including the use of atom-tracking and feed-forward methodologies, a combination of *layer-by-layer* data acquisition with post-data-acquisition correction procedures allows the additional correction of minor gradual lateral shifts due to an overall elastic bending of the tip apex, in the case of datasets in which the type of contrast remains largely undistorted. Simulations based on simplified pairwise potentials acting between model nonionic surfaces suggest that tip asymmetries may lead to appreciable distortions of atomic-scale force spectra that are absent when the surface force field is probed with symmetric tip apices. As such, it can be argued that the distortions in force-field maps that have been reported in the literature are due to irregularities associated with the probing tip apex, such as inherent asymmetry and/or extensive elastic response due to interaction forces, and are thus unlikely to represent intrinsic properties of the investigated sample surfaces. Since the general aim of the measurement of atomic-scale force fields is to study the physical and chemical properties of the sample surfaces in question rather than to probe artifacts, datasets exhibiting unusual changes in contrast patterns should ideally be disregarded. The use of post-data-acquisition correction procedures in suitable datasets, on the other hand, proves beneficial when addressing problems associated with drift and piezo creep, as well as the gradual lateral bending of asymmetric tip apices with decreasing tip–sample distance during the measurement of atomic-scale surface force fields.
